# Associations between Black and Mild Cigar Pack Size and Demographics and Tobacco Use Behaviors among US Adults

**DOI:** 10.3390/ijerph18126628

**Published:** 2021-06-20

**Authors:** Ollie Ganz, Jessica L. King, Daniel P. Giovenco, Mary Hrywna, Andrew A. Strasser, Cristine D. Delnevo

**Affiliations:** 1 Rutgers Center for Tobacco Studies, Rutgers Biomedical and Health Sciences, Rutgers University, New Brunswick, NJ 08901, USA; hrywnama@cts.rutgers.edu (M.H.); delnevo@sph.rutgers.edu (C.D.D.); 2 Department of Health & Kinesiology, University of Utah, Salt Lake City, UT 84112, USA; jess.king@utah.edu; 3 Department of Sociomedical Sciences, Columbia University Mailman School of Public Health, New York, NY 110032, USA; dg2984@cumc.columbia.edu; 4 Department of Psychiatry, University of Pennsylvania, Philadelphia, PA 19104, USA; strasse3@pennmedicine.upenn.edu

**Keywords:** tobacco regulatory science, cigars, tobacco control, tobacco packaging

## Abstract

Pack size is an important pricing strategy for the tobacco industry, but there is limited data on how users differ based on preferred pack size for cigar products. Using data from Wave 4 of the Population Assessment of Tobacco and Health Study, this study identified differences in adult cigar user characteristics based on pack size purchasing behavior among users of a top cigar brand, Black and Mild. Weighted chi-square tests were used to examine the associations between Black and Mild pack size and sociodemographic, cigar and other substance use characteristics. Overall, our study found that users of Black and Mild cigars differ by demographic, cigar and other tobacco use characteristics based on preferred pack size, with smaller packs appealing to younger, female, less-experienced and less-established smokers, and larger packs appealing to older, male, more experienced, and more dependent cigar smokers. Dual use of cigarettes and cigars was also higher among users of smaller packs. While this study is cross-sectional, findings suggest that minimum packaging laws for cigars may impact younger adults who are purchasing smaller pack sizes and likely experimenting with new cigar products and styles.

## 1. Introduction

In 2019, 3.6% of United States adults (over nine million) reported current cigar use; prevalence was highest among young adults (i.e., 18–44) and those who are non-Hispanic Black [1]. Cigars, defined as a roll of tobacco wrapped in leaf tobacco or another substance that contains tobacco [2,3], contain more chemicals than cigarettes, including tobacco-specific nitrosamines, exposing cigar smokers to higher concentrations of toxic and carcinogenic compounds than cigarettes [4]. As a result, cigar smoking is associated with increased all-cause mortality and increased risk of tobacco-related cancers, heart disease, and stroke, with daily users at even greater risk [4,5,6,7]. In the United States, health care expenditures attributable to exclusive cigar use near USD 284 million annually, with expenditures attributable to any cigar use nearing USD 1.75 billion annually [8]. Epidemiological research in the United States has recently begun to distinguish between cigar sub-types, finding that over 60% of current adult cigar users typically smoke mass-market cigarillos [2,3,9]. Given that these mid-sized cigars are generally used more frequently than large, premium cigars and are usually inhaled [10], they likely present elevated health risks and addictive liability. Therefore, decreasing cigar availability, appeal, and use—particularly for cigarillos—is a public health priority [11].

Cigars often cost less than cigarettes in the United States, which increases their appeal among youth, young adults, and individuals with lower income levels [12,13]. Indeed, a pack of cigars generally costs less than USD 2 in the United States, whereas the average price of a pack of cigarettes is USD 7.22 [14,15,16]. There are two primary drivers of this stark price difference; first, the products are taxed differently, with lower state and federal excise taxes for cigars versus cigarettes. Second, this difference can be explained by a lack of a federal minimum pack size mandate for cigars. While cigarettes in the United States are required to be sold in pack sizes of at least 20 [17] and the sale of single cigarettes is prohibited by the World Health Organization Framework Convention on Tobacco [18], there is no minimum pack size for cigars; as a result, cigars in the United States are sold in at least 12 different pack sizes ranging from single sticks to 60-packs, with smaller pack sizes becoming more popular in recent years [19,20]. Singles and two-packs can be sold as cheaply as USD 0.99 or less.

Pack size is a critical pricing strategy for the tobacco industry [21,22]. It is widely acknowledged in the cigarette literature that smaller pack quantities reduce barriers to use, as they are easier to conceal and carry and are less expensive than larger packs [22,23,24,25,26]. However, there is limited research on cigar purchasing behaviors, although some data suggest that smaller pack sizes are cheaper per pack [15,16]. Further, trial or cigar experimentation is associated with smaller pack sizes. On the other hand, tobacco companies have historically used larger pack sizes as a strategy to offer “free” cigarettes and to discourage switching to low-cost value brands [25]. Some studies suggest that consumption is partially driven by unit bias, such that consumers accustomed to smoking an entire pack will continue to do so regardless of the quantity in the pack [27]. Two studies examining the associations between cigar pack size and use found that those who purchased larger quantities smoked more [16,20].

The U.S. Food and Drug Administration (FDA), which has regulatory authority over the manufacturing, marketing, and distribution of cigars, could issue a product standard for cigar pack size [28], similar to the actions for the recently proposed flavored cigar ban [29], but has not done so to date. However, despite a limited evidence base for cigars, policies mandating minimum pack sizes for cigars have been enacted in over 200 municipalities, primarily in Massachusetts, Minnesota, California, and New York [30,31,32,33]. Preliminary analyses of the effects of the policies enacted in Boston and Minnesota indicate the policies reduced single cigar availability, increased cigar sale price, and reduced disparities in access across neighborhoods [32,33,34,35]. These findings suggest minimum pack size policies may accomplish the intended effects of reducing access to low-cost products. While the limited evidence thus far is promising, the impact of tobacco control policies is not always equitable [36,37,38,39]. In addition to differences in policy implementation across geographic areas, differential policy impact may occur based on which subgroups consume the product. For example, if younger, more price-sensitive individuals are more likely to purchase small pack sizes, a minimum pack size policy has the potential to reduce use among these subgroups, which are known to have elevated rates of use.

Identifying whether consumer characteristics differ by preferred pack size can inform which groups are most likely to be impacted by various minimum pack size regulations and can help to identify set points for minimum pack size laws that would deter use among less experienced smokers. Furthermore, identifying differences in pack size use may also inform our understanding of cigar product appeal based on how variations in pack size appeal to different consumers. To fill these gaps in the literature, the purpose of this study was to identify whether sociodemographic and smoking characteristics differ by pack size purchasing behavior using data from Wave 4 of the Population Assessment of Tobacco and Health (PATH) Study.

## 2. Materials and Methods

### 2.1. Data Source

The PATH Study is a national, longitudinal study of tobacco use risk factors and behaviors among non-institutionalized individuals in the United States, ages 12 and older [40,41,42]. This study used data from Wave 4 of the adult (ages 18 and older) public use data file. In Wave 4, a probability-based refreshment sample supplemented the longitudinal sample to account for attrition. This resulted in three types of adults in the Wave 4 sample: (1) longitudinal respondents, (2) respondents from the refreshment sample, and (3) adults who aged up from the youth cohort. Wave 4 data, when appropriately weighted, is able to produce nationally-representative, cross-sectional estimates. Details on sample design can be found elsewhere [41,42]. Briefly, Wave 4 data collection took place from December 2016 to January 2018 in-person via computer-assisted personal interview and audio-computer assisted self-interview [41]. This secondary analysis of deidentified, publicly available data received a non-human subjects determination from the Rutgers IRB.

### 2.2. Study Sample

We selected users of one brand—Black and Mild—for the present analysis for a number of reasons: Preference for tobacco brands varies greatly by consumer demographics and tobacco use characteristics (e.g., frequency of use) [13,40,41]. As a result, if more than one brand were included in the analysis, it would be difficult to disentangle differences in user characteristics based on brand preferences versus pack size preferences. Black and Mild was selected because it has been one of the most dominant brands in the cigar market over the last decade [19,43], and its product lines include a variety of traditional cigar, cigarillo, and filtered cigar products, which come in multiple pack sizes, including singles, 2–3 packs, 4–5 packs, and larger sizes. Additionally, Black and Mild cigars are used less frequently for blunts (i.e., when tobacco is removed and replaced with marijuana) compared with other brands [13], which helps to minimize the confounder of using cigars for marijuana use.

Black and Mild users were identified as those who reported that Black and Mild was their regular cigar brand or the last cigar brand that they used for at least one cigar type (i.e., traditional cigars, filtered cigars and/or cigarillos). Accordingly, adults who reported pack size for at least one Black and Mild cigar type were included in the analysis (*n* = 1088). However, the total analytic sample was 1253 since certain individuals reported smoking multiple cigar types (see Section 2.4 for more details).

### 2.3. Measures

#### 2.3.1. Dependent Variable

Pack size was assessed separately for each cigar product (i.e., traditional cigars, filtered cigars and/or cigarillos) among current users of each product who reported that they usually buy their cigar product in person, from the internet, or by telephone. These respondents were first asked, “Do you usually buy [cigar products] by the box or pack, or as single [cigar products]?” Those who selected “box or pack” were then asked, “How many [cigar products] come in the box or pack that you usually buy?” We categorized those continuous responses in two separate ways: for one analysis, we categorized these responses into (1) singles and 2–3 packs and (2) packs of 4 or greater, and for another analysis, we categorized responses into (1) singles and (2) packs of 2 or greater.

#### 2.3.2. Independent Variables

Demographic, cigar use and other tobacco/substance use correlates of Black and Mild pack size were examined. Demographic characteristics included: age (18–24, 25–44, 45 and older), sex (male, female), race/ethnicity (non-Hispanic white, non-Hispanic Black, non-Hispanic other and Hispanic), educational attainment (high school graduate or less, some college or greater), and annual household income (less than USD 25,000 per year, USD 25,000 to USD 49,000 per year, USD 50,000 per year or greater).

Cigar use variables included: smoking one’s first cigar of the day within 30 min of waking (continuous variable recorded as 30 min or less vs. greater than 30 min), initiation of cigar use prior to age 18 (yes or no), lifetime number of cigars smoked (fewer than 100, 100 or greater), number of cigars smoked per day (less than 1, 1, 2 or more), having a regular cigar brand, and length of time using one’s regular cigar brand among those reporting a regular brand (less than 2 years, 2 years or longer). Monthly cigar consumption (1 or fewer, between 1 and 4, between 4 and 20, greater than 20) was calculated by multiplying the number of cigars smoked per day by the number of days smoked per month. Users of multiple Black and Mild cigar types were asked each cigar use question separately for each cigar type, allowing for pack size and other cigar use variables to be examined separately by cigar type.

Other tobacco use included past 30-day use of cigarettes, electronic nicotine products, hookah and smokeless tobacco. Other substance use included past-year marijuana use and past-year blunt use. Past-year marijuana use was asked among those who reported marijuana use at any wave and who reported never having smoked a blunt. Past-year blunt use was asked among those who reported having ever heard of cigar products and had reported lifetime blunt use at any wave.

### 2.4. Analysis

For this study, we examined demographic, cigar use and other tobacco/substance use correlates of Black and Mild cigar pack size. Although pack size was assessed for all cigar types (i.e., traditional cigars, filtered cigars, cigarillos), we did not distinguish between cigar type in the analysis since all Black and Mild cigars on the market are what many would call “cigarillo-size.” While we did not distinguish between cigar type, we did consider whether individuals reported pack sizes for multiple Black and Mild cigar types. Of the 1088 Black and Mild cigar users who reported pack size for Black and Mild, the majority reported pack size for one type of Black and Mild cigar product (85%, *n* = 923). Among the 165 respondents who reported pack sizes for multiple Black and Mild cigar products, 145 individuals reported pack sizes for two different types of Black and Mild products (e.g., Black and Mild cigarillos and Black and Mild filtered cigars) and 20 individuals reported using all three types of Black and Mild products (i.e., Black and Mild cigarillos, Black and Mild filtered cigars and Black and Mild traditional cigars).

To ensure that these individuals’ data were captured for each cigar type, their records in the dataset were either duplicated or triplicated depending on how many Black and Mild cigar types were used. For example, if someone reported pack sizes for Black and Mild cigarillos and Black and Mild filtered cigars, they appeared in the dataset twice, with one record reflecting pack size and other relevant data for the cigarillos and another record reflecting pack size and other relevant data for the filtered cigars. In order to ensure that these individuals were not over-represented in the data after weighting, weights for these respondents were divided by two or three, depending on how many pack sizes they reported (e.g., weights were divided by three for those reporting pack sizes for all three types of Black and Mild cigars). This resulted in a total analytic sample of 1253 adults who used Black and Mild cigars.

Weighted chi-square tests were used to examine bivariate associations between Black and Mild pack size and demographic, cigar use and substance/other tobacco use characteristics. Pack size was examined in two different ways (see Section 2.3.1): (1) less than 4 vs. 4 or greater and (2) singles vs. packs of two or greater. Looking at pack size in multiple ways allowed for a more nuanced understanding of how users of smaller pack sizes may differ from one another, which could have implications for determining set points for minimum pack size laws. Data were weighted to be nationally representative and to account for non-response bias and oversampling. Variance estimation procedures were used to account for stratification and clustering utilized in sampling, and replicate weights, which were calculated using Fay’s variant of balanced repeated replication, were used to calculate standard errors. Cross-sectional weights were used, per the PATH user guide [40]. Analyses were conducted in Stata/MP Version 16.1 [44]. Data with denominators <50 or relative standard error >30% were suppressed.

## 3. Results

### 3.1. Characteristics of the Analytic Sample

The majority of Black and Mild smokers were male (67.79%) and had an annual household income of less than USD 25,000. Approximately half were ages 25–44 (51.99%), and a total of 55.82% of the sample completed high school or less. A total of 44.28% were non-Hispanic White, and 35.41% were non-Hispanic Black. The majority of Black and Mild smokers reported smoking their first cigar more than 30 min after waking (78.75%), initiating after the age of 18 (56.64%), smoking fewer than 100 cigars in their lifetime (77.81%) and having a regular cigar brand (83.05%). Among those with a regular brand, most reported having used that brand for 2 years or longer (65.52%). In terms of the number of cigars smoked per day and monthly consumption, the sample was evenly distributed across categories. About half of the sample used blunts in the past year (48.75%), and the most popular non-cigar tobacco product used in the past 30-days was cigarettes, which were used by the majority of the sample (70.66%). Three-quarters of those who use Black and Mild typically purchased small pack sizes (i.e., 1–3 packs), while a quarter purchased larger pack sizes.

### 3.2. Associations between Black and Mild Cigar Pack Size (Singles and 2–3 Packs vs. 4+ Packs) and Demographic, Cigar Use and Other Substance/Tobacco Use Characteristics

Bivariate analyses revealed that adults who use Black and Mild cigars differed in terms of age and sex based on cigar pack size when comparing users of singles and 2–3 packs vs. 4+ packs (Table 1). Specifically, those who usually purchased smaller pack sizes were disproportionately younger (32.60% ages 18–24, *p* < 0.001) and female (35.01%, *p* = 0.01) compared to those who usually purchased larger pack sizes (18.29% ages 18–24 and 23.67% female).

In terms of cigar use behaviors, a greater proportion of those who purchased larger pack sizes reported smoking their first cigar within 30 min of waking (27.09%; *p* = 0.045) compared with those who purchased smaller pack sizes (19.03%). Adults who purchased larger pack sizes also reported smoking a greater number of cigars in their lifetime, per day and per month, compared with those who purchased smaller pack sizes. Specifically, 40.44% of those who purchased larger pack sizes reported having smoked 100 or more cigars in their lifetime compared with 22.19% of those who purchased smaller pack sizes (*p* < 0.001). A total of 52.33% and 44.78% of those who purchased larger pack sizes reported smoking 2 or more cigars per day and more than 20 cigars per month, respectively, compared with 29.52% and 20.27% of those who purchased smaller pack sizes (*p* = 0.001 and *p* < 0.001). Among those who reported having a regular cigar brand, a greater proportion of those who purchased larger pack sizes reported having used that brand for two years or longer (74.08%) compared with those who purchased smaller pack sizes (58.90%; *p* = 0.018). Lastly, a greater proportion of those who purchased smaller pack sizes reported past 30-day cigarette use (73.25%), compared to those who purchased larger pack sizes (62.74%; *p* = 0.047).

### 3.3. Associations between Black and Mild Cigar Pack Size (Singles vs. 2+ Packs) and Demographic, Cigar Use and Other Substance/Tobacco Use Characteristics

Findings were similar when comparing users based on pack sizes of singles vs. packs of two or greater (Table 2). Users of singles were younger (31.95% ages 18–24 vs. 22.77% for packs of 2 or greater; *p* = 0.0099) and female (35.14% vs. 25.79% for packs of 2 or greater, *p* = 0.0149).

In terms of cigar use behaviors, a smaller proportion of those who purchased singles reported smoking their first cigar within 30 min of waking (18.64%; *p* = 0.0258) compared with those who purchased pack sizes of 2 or greater (26.32%) and having smoked 100 or more cigars in their lifetime (16.83% vs. 35.23% among users of packs of 2 or greater; *p* < 0.001). Consumption of cigars was greater among those who smoked two packs or greater compared with those who smoked singles; 34.53% of users of singles smoked fewer than one cigar per day, compared with 18.77% of users of the larger pack sizes (*p* < 0.001). A total of 30.82% of users of singles smoked fewer than one cigar per month, compared with 16.87% of users of the larger pack sizes (*p* < 0.001). Among users reporting a regular brand, users of singles were more likely to have used the brand for less than 2 years (41.03%) compared with users of larger packs (29.55%; *p* = 0.0429). Cigarette was more common among users of singles (74.17%) versus users of larger pack sizes (62.98%; *p* = 0.0120). The only unique finding compared with the findings in Table 1 is related to marijuana use. Past-year marijuana use was disproportionately common among users of singles (21.25%) compared with users of larger pack sizes (12.80%; *p* = 0.0321).

## 4. Discussion

This is the first study to characterize adults who use Black and Mild cigars, one of the most popular cigar brands in the United States, based on preferred pack size. We found that those who purchased larger pack sizes were older, more likely to be male and were more experienced and established cigar users with higher levels of dependence. Those who purchased smaller pack sizes were younger, less-experienced cigar smokers with lower daily and monthly consumption. These findings were consistent across two different definitions of smaller pack size; (1) singles and (2) singles and two to three packs. These findings align with King et al., who found in their longitudinal analysis of PATH data that larger pack size (box or pack vs. singles) was associated with smoking more cigarillos per day [15]. Similarly, in their review of previously secret tobacco industry documents aimed at examining how tobacco companies have used package quantity to target consumers, Persoskie et al. found that larger tobacco pack sizes were designed for heavier users, while smaller pack sizes were designed for newer and lighter users [22]. Our findings also align with studies that show that cigarillo users skew male and young and report lower levels of educational attainment and annual household income [2].

Smaller pack sizes are available at very low prices [16] and often feature price promotions [13], which may be why we found that they are preferred among younger individuals, who are historically a more price-sensitive group [45,46]. Cheaper cigars and smaller pack sizes also lower barriers to trial and experimentation [16] with the release of new products and brands, and historically have been designed to make trying and potentially switching to new brands more accessible to price-sensitive populations [2,5]. In other words, users of smaller pack sizes may be experimenting with different products, brands and styles and therefore buying fewer (and cheaper) cigars. This aligns with our finding that among those reporting a regular cigar brand, users of small pack sizes were less likely to be long-term users of that brand (2 years or longer), compared with users of larger pack sizes.

On the other hand, while larger pack sizes are more expensive overall, they are cheaper per stick and consequently may have better value for more frequent and established smokers [15,16]. Indeed, we found that users of larger pack sizes consumed more cigars per day and were more likely to smoke within 30 min of waking; therefore, larger pack sizes may be more economical for their consumption patterns. Additionally, those that smoke larger pack sizes seem to be more brand loyal. Given that they have likely “settled” on a preferred brand and are not inclined to experiment with others, they may seek to minimize repeated store visits by stocking up on their usual product. When examining differences in user characteristics by pack size, findings were almost the same when looking at singles and two to three packs vs. packs of four or greater, compared to looking at singles vs. all other pack sizes. Of note, two to three packs are often priced cheaply (e.g., USD 0.99) [19] and similarly to singles. This suggests that various set points for minimum packaging laws (e.g., banning the sale of singles, banning the sale of cigars in packs of fewer than four) would impact similar populations. One notable difference was that users of singles were more likely to report marijuana use compared with users of larger pack sizes, whereas this relationship was not significant when looking at users of singles and two to three packs vs. users of packs of four or greater. Concurrent use of marijuana and cigars is a common behavior, as some people believe that tobacco products enhance the effects of marijuana, or individuals use cigars to smoke marijuana in the form of blunts [47,48,49,50]; however, our study did not find differences in blunt use by pack size. Single cigars may be particularly appealing to marijuana smokers if they believe that tobacco products enhance the effects of marijuana and are looking for a cheap way to do so, particularly if these users are younger, as reported in our study, and therefore likely more price sensitive. However, marijuana and cigar co-use behaviors are complex, and more research is needed to understand the disproportionate use of marijuana among users of single cigars.

Overall, our study documented a high prevalence of cigarette use among all Black and Mild users, which reflects high rates of dual use among cigar users in general. Indeed, Corey et al. found that 58% of cigarillo smokers were also current, established cigarette smokers [2] and that 30% also used another tobacco product (i.e., non-cigar/non-cigarette product), demonstrating that poly-tobacco use is common among cigar users. Despite the high prevalence of cigarette use overall, we found that users of smaller pack sizes were more likely to be cigarette smokers compared to users of larger pack sizes. This is likely a reflection of the fact that smaller pack sizes are disproportionately purchased by younger people, who are more likely to use multiple products compared with older adults [1,51]. Additionally, cigarette smokers may be purchasing small packs of cigarillos as an alternative to cigarettes when they need to keep costs down. Sample size limitations prevented us from looking at cigarettes per day in our analysis, but future research should examine differences in cigarette consumption by cigar pack size among dual cigar and cigarette users. It is also worth noting that Black and Mild is owned by Altria, who may be cross promoting cigarette and cigar products via their extensive direct mailing lists and/or at the point-of-sale [52].

This study has limitations. First, the question about pack size in PATH is only asked among current cigar users, defined as smoking some days or every day, and non-current users who have smoked in the past 30 days, which may have prevented the inclusion of more recent initiates and infrequent cigar users in our analysis. Second, since our analysis only focused on one cigar brand, Black and Mild, findings are not necessarily generalizable to other cigar brands. Due to Black and Mild’s product offerings, we also did not examine differences by cigar type (e.g., traditional cigars, cigarillos, filtered cigars), but future research should assess pack size preferences among users of various cigar types, which may be important when considering minimum pack size policies [15,22]. Lastly, sample size limitations prevented us from examining more granular categorizations of pack sizes, which may have masked differences between users of certain pack sizes (e.g., singles vs. two-packs). More research is needed to replicate these findings across other cigar brands, types, and pack size categories.

## 5. Conclusions

Overall, our study found that users of Black and Mild cigars differ by demographic, cigar and other tobacco use characteristics based on preferred pack size, suggesting that smaller packs appeal to younger, less-experienced and less-established smokers, whereas larger packs appeal to older, more-experienced, and more-dependent cigar smokers. However, longitudinal data and studies with larger samples of cigar users reporting pack size are needed to understand how pack size preferences influence behavior trajectories. These data will be essential for revealing the potential impact of pack size on initiation, cessation and other patterns of use, including poly-tobacco use.

Since this study is cross-sectional, we are unable to estimate the potential impact of a minimum pack size policy on tobacco use behaviors—longitudinal, observational and experimental studies are needed for this type of evaluation. Nevertheless, our findings suggest that the implementation of minimum packaging laws (i.e., banning singles and other small pack sizes) for cigars—whether adopted by FDA via a product standard in the United States, or by way of the WHO FCTC on a global scale—may impact younger adults who are purchasing smaller pack sizes and likely experimenting with new cigar products and styles. While our study did not find differences in preferred pack size by race/ethnicity, overall use of cigars is disproportionately high among non-Hispanic, Black youth and adults [1,53]. Therefore, policies that restrict sales of cigar products, such as minimum pack size policies, could have a positive impact on reducing racial/ethnic tobacco use inequalities. However, it still remains unclear if minimum packaging laws would indeed reduce cigar consumption among users of smaller pack sizes, or if these individuals would instead purchase larger packs, potentially increasing their consumption [15]. Furthermore, such policies may not address use behaviors among more dependent and established users who prefer larger pack sizes, highlighting the need for multiple strategies to curb cigar use, such as taxation and cessation support.

## Figures and Tables

**Table 1:** Weighted demographic, cigar use and tobacco/substance use characteristics among Black and Mild adult cigar users, by pack size (singles and 2–3 packs vs. 4 packs or greater), from Wave 4 of the Population Assessment of Tobacco and Health Study, unweighted *N* = 1253.

	Singles or 2–3 Packs (*n* = 988)	4+ Packs (*n* = 265)	*p*-Value
	% (95% CI)	% (95% CI)	
	75.34 (71.51, 78.81)	24.66 (21.19, 28.49)	
Demographic characteristics			
Age (years) (*n* = 1253)			<0.001
18–24	32.60 (29.59, 35.76)	18.29 (13.25, 24.70)	
25–44	51.56 (47.27, 55.83)	53.29 (45.21, 61.21)	
45	15.84 (13.00, 19.16)	28.42 (21.97, 35.89)	
Sex (*n* = 1253)			0.0099
Male	64.99 (61.08, 68.72)	76.33 (68.77, 82.53)	
Female	35.01 (31.28, 38.92)	23.67 (17.47, 31.23)	
Race/ethnicity (*n* = 1253)			ns
Non-Hispanic white	41.78 (37.52, 46.18)	51.90 (40.57, 63.04)	
Non-Hispanic Black	37.69 (33.91, 41.62)	28.45 (22.19, 35.66)	
Non-Hispanic other	5.05 (3.72, 6.82)	4.39 (2.60, 7.32)	
Hispanic	15.48 (12.86, 18.52)	15.26 (6.37, 32.28)	
Educational attainment (*n* = 1246)			ns
High school graduate or less	56.44 (52.63, 60.18)	53.92 (42.58, 64.87)	
Some college or more	43.56 (39.82, 47.37)	46.08 (35.13, 57.42)	
Household annual income (*n* = 1200)			ns
Less than USD 25,000	57.08 (52.91, 61.15)	47.92 (36.79, 59.26)	
USD 25,000–USD 49,999	22.19 (19.19, 25.53)	24.93 (14.00, 40.39)	
USD 50,000 or greater	20.73 (17.39, 24.52)	27.15 (19.60, 36.30)	
Cigar use behaviors			
Smokes first cigar within 30 min of waking (*n* = 889)	19.03 (15.67, 22.91)	27.09 (19.87, 35.75)	0.0451
Initiated cigar use before 18 years of age (*n* = 842)	46.52 (41.35, 51.76)	33.76 (21.99, 47.95)	ns
Lifetime number of cigars smoked (*n* = 998)			<0.001
Fewer than 100	82.92 (79.79, 85.65)	59.56 (45.83, 71.95)	
100 or more	17.08 (14.35, 20.21)	40.44 (28.05, 54.17)	
Number of cigars smoked per day (*n* = 886)			0.0011
Fewer than 1	33.31 (29.38, 37.48)	18.45 (12.54, 26.32)	
1	37.18 (32.71, 41.87)	29.22 (18.63, 42.66)	
2 or more	29.52 (24.80, 34.72)	52.33 (40.83, 63.58)	
Number of cigars consumed per month (*n* = 882)			<0.001
Fewer than 1	29.89 (25.66, 34.50)	16.02 (10.11, 24.44)	
Greater than 1 but fewer than 4	26.26 (23.11, 29.68)	11.45 (7.27, 17.57)	
Greater than 4 but up to 20	23.58 (19.96, 27.63)	27.75 (20.34, 36.62)	
Greater than 20	20.27 (16.66, 24.42)	44.78 (37.93, 51.83)	
Has a regular cigar brand (*n* = 1253)	82.53 (79.29, 85.36)	84.66 (77.73, 89.72)	*ns*
Length of time with regular brand ^µ^ (*n* = 963)			0.0182
Less than 2 years	41.10 (36.94, 45.39)	25.92 (16.69, 37.94)	
2 years or longer	58.90 (54.61, 63.06)	74.08 (62.06, 83.31)	
Substance/other tobacco use			
Past-year marijuana use (*n* = 596) ^€^	20.60 (16.10, 25.98)	12.62 (7.26, 21.04)	0.0509
Past-year blunt use (*n* = 1139) ^€^	49.34 (44.67, 54.01)	46.80 (37.31, 56.53)	*ns*
Past 30-day cigarette use (*n* = 1253)	73.25 (69.05, 77.07)	62.74 (50.35, 73.66)	0.0474
Past 30-day electronic nicotine product use (*n* = 1251)	31.39 (27.60, 35.46)	27.54 (18.48, 38.91)	*ns*
Past 30-day hookah use (*n* = 1253)	13.42 (11.00, 16.27)	8.89 (5.51, 14.05)	0.0897
Past 30-day smokeless tobacco or snus use (*n* = 1251)	11.66 (9.31, 14.52)	13.76 (8.23, 22.10)	*ns*

^€^ Blunt users and marijuana users were mutually exclusive groups. ^µ^ Asked among those reporting a regular brand.

**Table 2:** Demographic, cigar use and tobacco/substance use characteristics among Black and Mild cigar users, by pack size (singles vs. packs of 2 or greater), from Wave 4 of the Population Assessment of Tobacco and Health Study, unweighted *N* = 1253.

	Singles (*n* = 889)	2+ Packs (*n* = 265)	*p*-Value
	% (95% CI)	% (95% CI)	
	68.62 (64.96, 72.06)	31.38 (27.94, 35.04)	
Demographic characteristics			
Age (years) (*n* = 1253)			0.0099
18–24	31.95 (28.80, 35.28)	22.77 (17.68, 28.82)	
25–44	51.94 (47.24, 56.61)	52.09 (44.77, 59.32)	
45	16.11 (12.98, 19.81)	25.14 (19.67, 31.54)	
Sex (*n* = 1253)			0.0149
Male	64.86 (60.79, 68.72)	74.21 (67.81, 79.71)	
Female	35.14 (31.28, 39.21)	25.79 (20.29, 32.19)	
Race/ethnicity (*n* = 1253)			ns
Non-Hispanic white	42.23 (37.81, 46.79)	48.76 (39.19, 58.42)	
Non-Hispanic Black	37.45 (33.57, 41.51)	30.94 (24.70, 37.97)	
Non-Hispanic other	4.43 (3.24, 6.02)	5.88 (3.52, 9.69)	
Hispanic	15.89 (13.12, 19.11)	14.41 (6.87, 27.78)	
Educational attainment (*n* = 1246)			ns
High school graduate or less	57.16 (53.32, 60.92)	52.90 (43.59, 62.00)	
Some college or more	42.84 (39.08, 46.68)	47.10 (38.00, 56.41)	
Household annual income (*n* = 1200)			ns
Less than USD 25,000	57.80 (53.63, 61.86)	48.38 (38.78, 58.12)	
USD 25,000–USD 49,999	21.81 (18.72, 25.26)	25.15 (15.87, 37.45)	
USD 50,000 or greater	20.39 (17.04, 24.21)	26.47 (19.38, 34.38)	
Cigar use behaviors			
Smokes first cigar within 30 min of waking (*n* = 889)	18.64 (15.10, 20.42)	26.32 (20.42, 33.20)	0.0258
Initiated cigar use before 18 years of age (*n* = 842)	46.69 (41.54, 51.92)	35.81 (25.50, 47.62)	0.0877
Lifetime number of cigars smoked (*n* = 998)			<0.001
Fewer than 100	83.17 (79.78, 86.09)	64.77 (52.73, 75.19)	
100 or more	16.83 (13.91, 20.22)	35.23 (24.81, 47.27)	
Number of cigars smoked per day (*n* = 886)			<0.001
Fewer than 1	34.53 (30.49, 38.80)	18.77 (13.32, 25.78)	
1	37.76 (32.98, 42.78)	29.57 (20.78, 40.19)	
2 or more	27.72 (23.03, 32.95)	51.66 (43.07, 60.15)	
Number of cigars consumed per month (*n* = 882)			<0.001
Fewer than 1	30.82 (26.53, 35.46)	16.87 (11.65, 23.78)	
Greater than 1 but fewer than 4	26.74 (23.34, 30.43)	13.63 (9.59, 19.02)	
Greater than 4 but up to 20	23.59 (19.72, 27.96)	26.77 (20.51, 34.12)	
Greater than 20	18.86 (15.40, 22.87)	42.74 (36.13, 49.61)	
Has a regular cigar brand (*n* = 1253)	82.78 (79.31, 85.77)	83.65 (77.09, 88.60)	ns
Length of time with regular brand ^µ^ (*n* = 963)			0.0429
Less than 2 years	41.03 (36.71, 45.50)	29.55 (20.93, 39.93)	
2 years or longer	58.97 (54.50, 63.29)	70.45 (60.07, 79.07)	
Substance/other tobacco use			
Past-year marijuana use (*n* = 596) ^€^	21.25 (16.40, 27.06)	12.80 (7.85, 20.18)	0.0321
Past-year blunt use (*n* = 1139) ^€^	48.86 (43.91, 53.84)	48.50 (40.29, 56.78)	*ns*
Past 30-day cigarette use (*n* = 1253)	74.17 (69.70, 78.19)	62.98 (52.84, 72.09)	0.0120
Past 30-day electronic nicotine product use (*n* = 1251)	31.56 (27.64, 35.75)	28.01 (20.13, 37.54)	0.04657
Past 30-day hookah use (*n* = 1253)	13.19 (10.76, 16.07)	10.36 (7.02, 15.03)	ns
Past 30-day smokeless tobacco or snus use (*n* = 1251)	11.74 (9.30, 14.72)	13.13 (8.76, 19.24)	ns

^€^ Blunt users and marijuana users were mutually exclusive groups. ^µ^ Asked among those reporting a regular brand.

## Data Availability

Data used in this study are publicly available. United States Department of Health and Human Services. National Institutes of Health. National Institute on Drug Abuse, and United States Department of Health and Human Services. Food and Drug Administration. Center for Tobacco Products. Population Assessment of Tobacco and Health (PATH) Study (United States) Public-Use Files. Inter-university Consortium for Political and Social Research (distributor), 21 October 2020. https://doi.org/10.3886/ICPSR36498.v11.
